# In Vitro Infrared Thermographic Assessment of Temperature Change in the Pulp Chamber during Provisionalization: Effect of Remaining Dentin Thickness

**DOI:** 10.1155/2020/8838329

**Published:** 2020-11-10

**Authors:** Mariusz Lipski, Krzysztof Woźniak, Liliana Szyszka-Sommerfeld, Mariusz Borawski, Agnieszka Droździk, Alicja Nowicka

**Affiliations:** ^1^Department of Preclinical Conservative Dentistry and Preclinical Endodontics, Pomeranian Medical University, Szczecin, Poland; ^2^Department of Orthodontics, Pomeranian Medical University, Szczecin, Poland; ^3^West Pomeranian University of Technology, Szczecin, Poland; ^4^Department of Integrated Dentistry, Pomeranian Medical University, Szczecin, Poland; ^5^Department of Conservative Dentistry and Endodontics, Pomeranian Medical University, Szczecin, Poland

## Abstract

Interim crowns and partial fixed dental prosthesis materials generate exothermic heat during polymerization. The amount of heat transmitted to the pulp chamber can be a function of several factors, including the thickness and quality of the remaining dentin after crown preparation. The aim of this in vitro study was to measure with infrared thermography the temperature changes on the adjacent surface of the chamber roof of premolar teeth extracted from young and old patients (having different thicknesses of remaining dentin after crown preparation) during fabrication of provisional resinous restorations. Twenty extracted human first and second maxillary premolar teeth (10 from young patients, with a relatively large pulp chamber, and 10 from older patients, with a relatively small pulp chamber) were used. The roots were sectioned to expose the inner side of the chamber roof, and the crowns were provisionalized after preparation for a metal-ceramic crown. Two provisional materials, Turbo Temp 2 and Luxatemp Fluorescence, were used. Temperature changes on the inner side of the chamber roof were measured at 2-second intervals using an infrared thermal imaging camera. After completion of the temperature recordings, the teeth were sectioned and the remaining dentin thickness was determined. The older group (mean thickness: 2.82 mm) and younger group (mean thickness: 1.9 mm) differed significantly in dentin thickness (*P* < 0.014). The mean greatest temperature increases recorded on the chamber roof of teeth with less remaining dentin were 4.07°C for Turbo Temp 2 and 3.94°C for Luxatemp Fluorescence, while increases in the premolars with greater dentin thickness were 1.69°C for Turbo Temp 2 and 1.64°C for Luxatemp Fluorescence. Significant interactions were found between tooth groups (*P* < 0.000001for Turbo Temp 2 and for Luxatemp Fluorescence). No significant differences were found between assessed materials regardless of the thickness of the remaining dentin (*P* > 0.38for the older group and *P* > 0.29 for the younger group). Dentin had a significant effect in limiting the temperature increase generated during polymerization of provisional materials, indicating good thermal insulating properties of this tissue. A remaining dentin thickness of 1.9 mm or more is sufficient to protect the pulp from any temperature increase during provisionalization using tested materials.

## 1. Introduction

Interim crowns are essential in treatment with partial fixed dental prosthesis. Their purpose is stabilization of occlusion, protection of the teeth and periodontal tissues, and maintenance of function while the final restoration is being fabricated [1–4]. Acrylic and bis-acrylic resins are widely used for direct temporary restorations [1–5]. When fabricating interim crowns and partial fixed dental prosthesis using a direct technique, heat generated by the exothermic reaction of polymerizing resins may cause thermal damage to the vital pulp [6]. Zach and Cohen [7] reported that a 5.5°C intrapulpal temperature rise in rhesus macaques caused 15% of the pulps to lose vitality. When the intrapulpal temperature rose by 11.1°C, 60% of the teeth tested exhibited necrosis, and a 16.6°C increase led to necrosis of the pulp in 100%. However, Baldisara et al. [8] in experiments on human teeth found that average increases of 11.2°C did not damage the pulp, with no signs of inflammation or reparative processes within 2-3 months after heating.

The amount of heat transmitted to the pulp chamber can be a function of several factors, including the chemical composition of the provisional material used and its volume [9–13], type of matrix used [14], and thickness and quality of remaining dentin after crown preparation [15–18]. The correlation between dentin thickness and heat transfer was described in studies investigating temperature increases generated during photopolymerization of composite resins [19–23], root canal filling with thermoplasticized gutta-percha [24, 25], ultrasonic removal of posts [26], ultrasonic retrieval of intracanal separated files [27], and ultrasonic root planing and scaling [28, 29], even more strongly supporting the observation of the isolating properties of the dentin.

The aim of this in vitro study was to measure with infrared thermography the temperature changes on the adjacent surface of the chamber roof of premolar teeth extracted from young and old patients (teeth with different remaining dentin thicknesses after crown preparation) during fabrication of provisional resinous restorations. The null hypothesis considered in this study was the lack of temperature differences in the pulp chamber of the premolars removed from older patients with higher remaining dentin thickness in comparison with premolars removed from younger patients with lower remaining dentin thickness during the procedure described above.

## 2. Material and Methods

### 2.1. Sample Preparation

**Figure 1:**
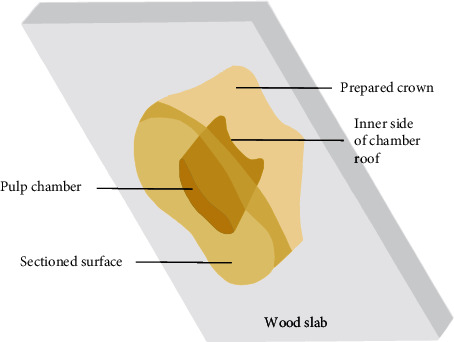
Schematic drawing of fixation of prepared tooth during provisionalization and temperature measurement.

Twenty intact extracted human first and second maxillary premolar teeth were used in this study. Ten teeth were extracted for orthodontic reasons from young patients (teeth with a relatively large pulp chamber), and 10 teeth were extracted for periodontal reasons from older patients (teeth with a relatively small pulp chamber). Ethical approval was gained from the Pomeranian Medical University Ethics Committee. The roots were sectioned with a carboround disk approximately 2 mm below the cementoenamel junction perpendicular to the long axis of the tooth. The pulpal chambers were cleaned of pulp remnants to expose the inner side of the chamber roof. Then the crowns were fixed in a wood slab with the whole entire crown surface exposed to the air (Figure 1) and impressions were taken with silicone putty (Star VPS Putty, Danville Materials, San Ramon, Calif). Impression trays were made from trimmed 10 ml Monoject syringes to achieve an identical volume of impression material during fabrication of each crown. Another silicone index was fabricated and cut longitudinally for ideal tooth reduction. The teeth were prepared for a metal-ceramic complete crown with a 1.5 mm shoulder. Preparations were completed with diamond fissure and conical burs. Two materials based on bis-methacrylate were tested: Turbo Temp 2 (Danville Materials, San Ramon, Calif) and Luxatemp Fluorescence (DMG, Hamburg, Germany). The resin materials were mixed according to the manufacturer's instructions and introduced into the impression, which was then positioned on the prepared tooth. After complete polymerization of the resin, the tray and resin crown were removed.

### 2.2. Temperature Measurement

To obtain resin crown fabrication and temperature measurements, the wood slabs were mounted onto a metal fork. Temperature changes were recorded using a ThermaCam SC500 thermal imaging camera (Flir, Danderyd, Sweden) and its dedicated software package. The camera was mounted on a stand perpendicular to the inner side of the chamber roof at a distance of 15 cm. The thermograms were recorded at 2 s intervals over a period of 6 min. The experiment was carried out under controlled environmental conditions (ambient temperature = 20 ± 1.8°C, humidity = 50 ± 5%, and air flow < 0.5 m/s). The camera was calibrated for distance, ambient temperature, and emissivity of the root tissue. The emissivity is defined as the ratio of power radiated by a substance to the power radiated by a blackbody at the same temperature and depends on the nature of the radiating surface. Values range from 0, for a perfect reflector that does not radiate at all, to 1, for a blackbody. In this study, the emissivity of the root tissues was calculated to be 0.91 using the method described by Kells et al. [30]. The temperature rise was calculated by taking the maximum temperature of the heated inner side of the chamber roof and subtracting the ambient temperature. The temperature of heated tooth tissues was allowed to return to ambient temperature in between repetitions.

### 2.3. Remaining Dentin Thickness Assessment

After completion of the temperature recordings, the teeth were sectioned, and the remaining dentin thickness was determined. The lowest distance between the pulp chamber and prepared occlusal surface was measured to the nearest 0.01 mm by using an electric digital caliper (Orthopli Corp., Philadelphia, Pa) with the aid of a surgical operating microscope (Optilion PICO LED Microscope, Seliga Microscopes, Łodź, Poland).

### 2.4. Statistical Analysis

The normal distribution of data was confirmed by the Shapiro–Wilk test. Student's *t*-test was used to compare the results between the groups. The number of teeth *n* = 10 in each group was sufficient to detect with 80% probability significant difference between groups in temperature increase equal to 1.1°C, assuming that the standard deviation of the increase is 0.8°C.

## 3. Results

Table 1 shows values for the thickness of the remaining dentin and temperature increases (mean ± standard deviation and range) recorded on the adjacent surface of the roof of the pulp chamber. A significant difference was found between the dentin thickness of the older group (mean thickness: 2.82 mm) and the younger group (mean thickness: 1.9 mm) (*P* < 0.014). The mean greatest temperature increases recorded on the chamber roof of teeth with less remaining dentin were 4.07°C for Turbo Temp 2 and 3.94°C for Luxatemp Fluorescence, while increases in the premolars with greater dentin thickness were 1.69°C for Turbo Temp 2 and 1.64°C for Luxatemp Fluorescence. Significant interactions were found between tooth groups (*P* < 0.000001for Turbo Temp 2 and for Luxatemp Fluorescence). No significant differences were found between assessed materials regardless of the thickness of the remaining dentin (*P* > 0.38 for the older group and *P* > 0.29 for the younger group).

**Figure 2:**
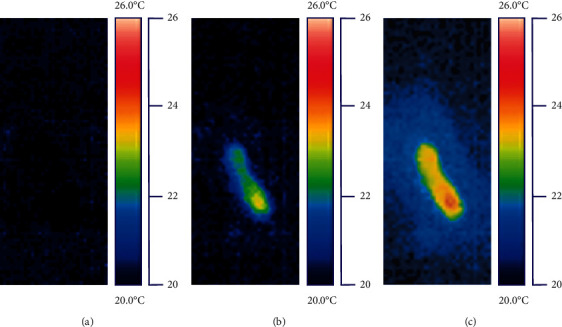
Selected thermograms of the inner side of chamber roof of premolar with lower dentin thickness (younger group) recorded during provisionalization using Turbo Temp 2. (a) Thermogram recorded directly after positioning of impression tray on tooth. (b) Thermogram recorded 30 s after positioning; temperature increase was 2.8°C. (c) Thermogram recorded 60 s after position; temperature increase was 3.9°C.

Figure 2 presents selected thermograms of the representative premolar recorded during provisionalization using Turbo Temp 2.

## 4. Discussion

This in vitro study investigated the temperature increases in the pulp chamber during direct fabrication of provisional restorations using 2 commercially available bis-acrylic-based materials in teeth with different remaining dentin thicknesses after crown preparation. The results of this study indicated that the differences in the temperature increases between premolars with higher dentin thickness in comparison with teeth with lower dentin thickness were significant; therefore, the data support the rejection of the null hypothesis. It has been suggested that a temperature increase of 5.5°C, when transmitted to the pulp chamber, could be responsible for damage to the pulp tissue [7]. With the experimental technique applied in this study, the temperature increases on the adjacent surface of the pulp chamber of teeth with either thin or thick remaining dentin never exceeded 5.5°C.

The findings of this study are in agreement with those of Chiodera et al. [10], who reported temperature increases of 3.4–5.5°C when the provisional resin materials were encored in a putty matrix. When a polyvinyl matrix was used, the temperature increased by 4.0–8.2°C. Akova et al. [14] investigated the effect of different desensitizers on pulpal temperature increases during provisionalization and found a temperature ranging from 3.5 to 6.2°C. However, in other in vitro studies, Usumez et al. [11] compared a resin-based dentin desensitizer and polyurethane cyanoacrylate adhesive and found temperature increases significantly exceeding 5–6°C, and Kim and Watts [9] evaluated the exothermic reaction of the mono- and dimethacrylate-based materials and recorded temperature increases ranging from 5.1°C to 12.7°C. Differences in methods used in these studies can explain this variability in results.

In the present study, the temperature changes were determined in premolars removed from older patients that had a remaining dentin thickness of 2.82 mm after preparation in comparison with premolars removed from younger patients with a remaining dentin thickness of 1.9 mm. The mean temperature increase recorded in young teeth was slightly more than twice that in teeth extracted from older patients. These results indicated that thermal transfer from the outside crown surface to the pulp chamber is affected by dentin thickness and agree with the findings of Altintas et al. [18], who reported significantly lower temperature increases with thicker dentin specimens. In that study, the provisional materials induced temperature increases of 1.7–3.6°C for a 2 mm dentin disc and 2.9–5.0°C for 1 mm.

This study involved young and old teeth. The physical and chemical characteristics of old dentin differ from those of young dentin. Old dentin is more mineralized than young dentin, with aging in dentin occurring as changes such as the formation of peritubular dentin on the inner walls of dentinal tubules leading to complete obliteration of tubules [15]. These differences between young and old dentin may have implications for temperature increases, but no studies to date have assessed the influence of tooth age on the thermal conductivity of dentin tissue. However, Fanibunda and Sa [16] found that caries-affected dentin had a significantly higher thermal conductivity, i.e., lower thermal insulating properties, compared to normal dentin. Tosun et al. [17] reported a similar conclusion after comparing the temperature rise with normal and carious primary tooth dentin during photopolymerization of resin-containing materials. This finding suggested that thermal isolating properties may be affected not only by thickness of dentin but also by tissue quality, which might have influenced the results in this study.

The polymerization of provisional materials is an exothermic process with the extent of excursion dependent on the composition of the resin. Michalakis et al. [12] compared polymethyl methacrylate (Jet), polyvinylethyl methacrylate (Snap), bis-acrylic composite (Protemp II), and urethane dimethacrylate (Revotec) with respect to their exothermic reaction properties during polymerization. Polymethyl methacrylate produced the highest exothermic reaction; polyethyl methacrylate, polyvinylethyl methacrylate, and bis-acrylic resin were not significantly different from each other. Lieu et al. [13] compared peak temperatures during polymerization of 5 provisional resin materials. The increases in temperature of self-curing resins (Integrity, Protemp) were significantly greater than those of dual-cure resins (Iso-Temp, TCB Dual Cure, Provipont DC). In the present study, Turbo Temp 2 and Luxatemp Fluorescence materials were used for the fabrication of provisional crowns, and no difference was found between the temperature increases they generated. This result can be explained by the lack of compositional difference; Turbo Temp 2 and Luxatemp Fluorescence are bis-acryl-based materials.

Previous thermal studies of fabrication of provisional restoration used thermocouples to measure the temperature inside the pulp chamber [9–13, 18]. In the present study, a thermal imaging camera was used to measure temperature increases on the adjacent surface of the pulp chamber. The camera determined the temperature over the whole area studied (in this study adjacent surface of the chamber roof) rather than at a selected point of contact, as occurs with the thermocouple. In this way, the recording of a maximum temperature can never be overlooked with the use of a noncontact infrared camera [24–26]. One of the limitations of this study is that in vitro simulation of the clinical situation is difficult because of the absence of blood flow in the pulp and fluid motion in the dentinal tubules. Also, in vitro study prevents dissipation of the generated heat, causing overestimation of the real temperature in the pulp chamber. Finally, the surrounding periodontal tissues can promote heat convection in vivo, limiting the pulpal temperature increase [18, 26, 30]. Thus, the results of in vitro studies may not be directly transferred to the clinical situation, but certain relevant conclusions are possible, as described below.

## 5. Conclusions

Within the limitations of this in vitro study, the following conclusions can be made. First, dentin had a significant effect in limiting the temperature rise generated during polymerization of provisional materials, indicating the good thermal insulating properties of this tissue. Second, a remaining dentin thickness of 1.9 mm or more is sufficient to protect the pulp from any temperature increases during provisionalization using bis-acryl-based materials (Turbo Temp 2 and Luxatemp Fluorescence).

## Figures and Tables

**Table 1:** Thickness of remaining dentin and temperature increases (mean, standard deviation, and range) recorded on the entire surface of the chamber roof during fabrication of provisional restoration.

Group	Remaining dentin thickness (mm) Mean (SD) min/max	Temperature rise (°C) Mean (SD) min/max	
		Turbo Temp 2	Luxatemp Fluorescence
Older	2.82 (0.76) 1.7/3.9	1.69 (0.41) 1.1/2.4	1.64 (0.54) 0.9/2.7
Younger	1.75 (0.75) 0.9/3.1	4.07 (0.99) 1.9/5.2	3.94 (0.82) 2.6/4.9

## Data Availability

The data used to support the ﬁndings of this study are available from the corresponding author upon request.
